# Tiger abundance and density estimates from non-invasive sources: Camera-trap and genetic capture-recapture analyses

**DOI:** 10.1371/journal.pone.0361369

**Published:** 2026-11-04

**Authors:** Kanchan Thapa, Marcella J. Kelly, Lisette Waits, Manisha Bista, Jivan Shakya, Maheshwar Dhakal, Naresh Subedi, Samundra Ambuhang Subba, Netra Sharma Sapkota, Dibesh Karmacharya

**Affiliations:** 1 Department of Fish and Wildlife Conservation, Virginia Tech, Blacksburg, Virginia, United States of America; 2 WWF Nepal, Baluwatar, Kathmandu, Nepal; 3 Laboratory for Ecological, Evolutionary and Conservation Genetics, Department of Fish and Wildlife Sciences, University of Idaho, Moscow, Idaho, United States of America; 4 Center for Molecular Dynamics Nepal, Thapathali, Kathmandu, Nepal; 5 Ministry of Agriculture, Forests and Environment, Babarmahal, Kathmandu, Nepal; 6 National Trust for Nature Conservation, Kathmandu, Nepal; 7 Department of Wildlife and Natural Resources, Tarleton State University, Stephenville, Texas, United States of America; 8 Independent Researcher, Kathmandu, Nepal; 9 School of the Environment, University of Queensland, St. Lucia, Queensland, Australia; Amity University, INDIA

## Abstract

We present tiger density and abundance estimates derived independently from two non-invasive sampling techniques: camera-trapping and fecal genetic sampling in same geographic areas. Camera-trap data collected in 2009 covered 359.2 km^2^ of lowland core area of Chitwan National Park using 187 camera-trap locations and 2,821 trap-nights obtaining 264 identifiable photographs of 41 tigers with 103 recaptures. Fecal genetic sampling in 2011 surveyed 325 km^2^ along three spatial routes covering 1,173 km and collecting 257 tiger fecal samples. An eight multi-locus genotyping assay identified 31 tigers with 42 recaptures. We analyzed each dataset separately using spatially explicit capture-recapture models under Maximum likelihood and Bayesian framework. Maximum Likelihood-based density estimates from DENSITY were 2.55 (SE 0.6) tigers per 100 km² for camera traps and 2.57 (SE 0.9) for genetic data, while Bayesian estimates from SPACECAP were slightly lower at 2.4 (SE 0.3) and 2.2 (SE 0.5), respectively. Maximum Likelihood abundance estimates were 66 (SE 7.9) for camera data and 88 (SE 19.3) for genetic data, whereas Bayesian estimates were 64 (SE 7.9) and 93 (SE 19), respectively. Two independent data sources broadly comparable density estimates, though uncertainty was greater for the genetic dataset, which included fewer identified individuals and spatial recaptures. Because the data were surveyed in different years and differed in sampling effort, differences in precision cannot be attributed solely to sampling method. Non-invasive genetic sampling nonetheless produced density and abundance estimates broadly consistent with an established camera trap protocol, suggesting that integrating the data sources can enhance robust tiger monitoring and inform effective conservation planning.

## Introduction

Knowing population abundance or density of wildlife populations is central to their conservation and management [1,2]. For rare and cryptic animals, the answer to “how many?” is often unknown [3]. Making an objective decision regarding the conservation status of an animal population, especially for rare and cryptic species, requires precise estimates using robust analytical techniques, which must consider imperfect ability to detect species and individuals. Non-invasive sampling techniques that may use, for example, conventional traps, automated cameras, fecal DNA, and hair snares have been widely used within a capture-recapture (CR) framework [4] to estimate population parameters such as abundance and density for many species. There has been widespread use of traditional, non-spatial techniques to assess populations of elusive and sparsely distributed animals, especially big cats such as tigers (*Panthera tigris tigris*: [5]), leopards (*Panthera pardus*: [6]), snow leopards (*Panthera uncia*: [7]), jaguars (*Panthera onca*: [8]), and pumas (*Puma unicolor*: [9]). Because of the shortcomings of these traditional CR models, spatially explicit capture-recapture (SECR-Maximum Likelihood (ML) or SECR-Bayesian (B)) models have been developed that more directly incorporate how animals are detected by a trap array, conditioned on the ecological processes governing their distribution and movement in space into the detection process [10–13].

Camera-trapping has proven an efficient tool for monitoring rare and cryptic animals, including tigers [14], and in determining the conservation status of species at risk [15]. Multiple studies have shown the utility of non-invasive approaches using camera-trapping for estimating population size [6,16], occupancy [17], habitat use [18], and for studying carnivore communities [19–21]. In addition, non-invasive genetic sampling has become a powerful tool for studying and monitoring elusive and low-density species, including using genetic sampling in a capture-recapture framework [22,23]. Additionally, non-invasive genetic sampling using scats can provide useful insight into genetic diversity and connectivity [23–27], and into the feeding ecology of the species under study [28]. Both camera and genetic techniques provide an avenue for researchers to collect in-depth population monitoring information [29] and provide a growing analytical framework in which to advance our sampling and analytic methodologies [10,12,30–33], complementing and extending our efforts for better understanding species biology. Recently, there has been growing interest in providing improved estimates of population parameters from a single or multiple sources or reanalysis of data using the recently developed spatial, capture-recapture models [6,16,34] to better inform conservation of rare and cryptic species.

The Terai Arc Landscape (TAL) holds a meta-population of tigers across the foothills of the Himalayas in Nepal and northwest India [35]. Population monitoring of tigers using camera-trapping has been conducted at the multiple sites across the TAL in Nepal and India [36]. A limited number of studies have applied molecular genetic techniques to estimate tiger population abundance and density in India [30,37]. In contrast, non-invasive genetics approach in Nepal has been applied primarily to snow leopards [34,38,39], with no comparable studies conducted for tigers across the TAL. The Nepal Tiger Genome Project pioneered non-invasive genetic methods using tiger scat to generate genetic data sets to inform conservation genetics and population assessment of tigers in the TAL [24].

We estimated tiger density and abundance independently from camera-trap and non-invasive genetic data using SECR models. Because the two datasets were collected in the sampling area but in different sampling periods (2009 and 2011), we used the comparison to assess the consistency of SECR-derived population estimates across sampling approaches and periods rather than to directly evaluate the relative performance of the two methods. Secondly, we estimated tiger density separately from both data sources employing spatial capture-recapture (SECR) approaches that consider heterogeneity in detection probability arising from different exposure of individuals to the trap array, thereby more explicitly linking spatial trap arrangement into the estimation process. This study also demonstrates how non-invasive sampling can be used in population monitoring of an endangered, elusive, flagship species.

## Materials and methods

### Ethics statement

All the tiger scat samples were collected non-invasively without capturing or handling any animal. The study permit was granted by the Department of National Parks and Wildlife Conservation, Ministry of Forest and Soil Conservation, Government of Nepal, Kathmandu, Nepal (Letter No. 403–08/09/2010). Furthermore, Animal care and use committee approval was not required as it was based on the secondary data that used non-invasive techniques such as photographs.

### Study Area

This study was carried out in Chitwan National Park (CNP, 27°30′N, 84°20′E) located in central Nepal. The park covers an area of 932 km^2^ in the sub-tropical lowland forest of the inner Terai. The sampling area within the park covers the core area of the lowland forest south of the Rapti and Narayani Rivers and covers an area measuring between 421 km^2^ (Fig 1). CNP is contiguous with the Valmiki Tiger Reserve, India, to the south, and the Parsa National Park, Nepal to the east. CNP represents a source population of tigers in the eastern part of the TAL [24]. The major habitat types of CNP and surrounding areas include grassland (176 km^2^), forest (2,957 km^2^), and settlement and agricultural areas (1,312 km^2^). CNP has three distinct seasons: winter, summer, and the monsoon season [16]. The mean annual rainfall is 2,437 mm per year, with highest rainfall during the months of July-September [16]. Average ambient temperature varies from a minimum of 11^◦^C in winter to a maximum of 38^◦^C in summer season. Tigers (*Panthera tigris tigris*), leopards (*Panthera pardus fusca*), and wild dogs (*Cuon alpinus*) are the top carnivores, and the large ungulates include pachyderms such as the greater one-horned rhinoceros (*Rhinoceros unicornis*) and Asian elephant (*Elephas maximus*). Gaur (*Bos gaurus*), sambar (*Rus unicolor*), spotted deer (*Axis axis*), barking deer (*Muntiacus muntjac*), wild boar (*Sus scorfa*), hog deer (*Axis porcinus*), and primates are the potential carnivore prey found in CNP. More details on the CNP are available elsewhere [16,40].

**Fig 1:**
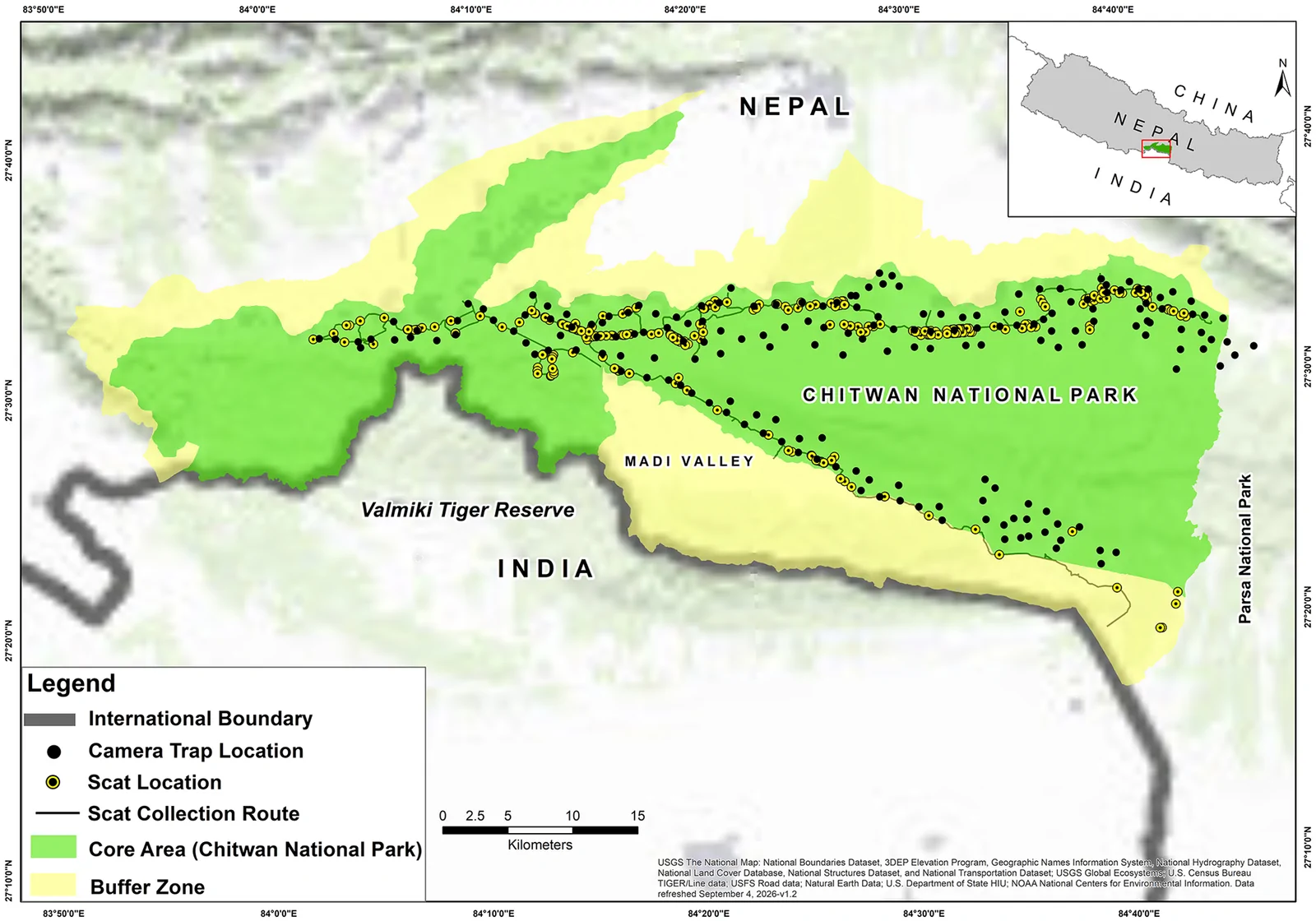
Study area showing camera-trap locations (black dots) and scat location (yellow dots) along with search routes (black lines) across the lowland forest in core area of Chitwan National Park.

### Camera-trap survey

We selected a subset of data from the 2009 camera-trap survey [41] covering 359.2 km^2^ of the low-land core area of Chitwan National Park. Within the core area, we followed standard procedures for conducting camera-trap surveys [42]. We divided the lowland area into 8 blocks, each measuring an average of 38 km^2^ (SD 10.18), for conducting the camera-trap survey based on previous informal records and regular site-specific tiger monitoring conducted by CNP staff. First, we did an extensive survey of the blocks to identify strategic locations for installing camera traps. Based on indirect signs and current movements of animals, we chose 187 locations with an average of 24 locations per block (SD = 4.0). To maximize capture probability, we positioned our camera-trap locations within habitat features known as tiger travel routes, such as forest roads, dry stream beds, and human and animal trails [14]. The average inter-trap distance between two consecutive locations was approximately 1.5 km (SE 0.41). We used a combination of 2 passive remote cameras (Moultrie Feeders and Stealth Cam, USA) at each location that were activated by animal movement and placed on either side of the trail [42]. We collected other ancillary information, such as date, time, and spatial location of the camera-traps for use in our analysis. We simultaneously worked in three blocks at a time, and each location was sampled for 24 hours for 16 consecutive days in each block. After 16 days, the cameras were moved to the next block until all survey blocks were completed. Our camera-trap survey was conducted in the winter season from December 2008 to March 2009.

### Fecal DNA survey

The fecal DNA survey was carried out in 324.9 km^2^ of the core area of the national park as a part of Nepal Tiger Genome Project (2011−14) [24]. We used Mondol et al. [37] field-sampling design for collection of fecal samples with modifications. Due to the linear orientation of the study area, we divided the core area of the national park into three blocks: Sauraha, Madi, and Tiger Tops. We selected three spatial routes, embedded within the earlier camera-trap survey, covering an average length of 65.13 (SD 9.20) km within each block, ensuring that all the scats in the areas have high probability of detection [16]. The spatial routes consisted of firelines, motorable roads, and human and animal trails, which have high probability of use by tigers [14]. Three observers walked across each spatial route searching for scat samples. Three observers sampled each route continuously for six days, rested on the seventh day and returned to the starting location of the route to begin the search over again. Each sampling occasion, therefore, spanned six days, ensuring that scats were fresh, less than seven days old [37]. The total sampling period was 42 days, consisting of six sampling occasions and sampling effort was 1173 km of routes walked. We conducted a survey in the winter season in December 2011.

Upon encountering scats in the field, we scratched a few grams from upper and side surfaces to obtain samples [24,37]. We stored the tiger scat samples in 2-ml vials at a 1:4 ratio filled with dimethyl sulphoxide saline solution (DETs buffer) [24,43,44]. We also took a 200–300 g sample from the widest portion of the scat for future molecular diet study, and the rest of the scat was left in the original location to avoid possible change in behavior due to the territorial nature of tigers. To avoid double-collection of the same scats in subsequent sampling sessions, we either marked the scat with a small red dot by nail polish (in case of dried samples) or pushed a twig inside the substrate at the side of the scat (in case of wet scats) after collecting the samples.

DNA was extracted from scat samples using a commercially available DNA stool kit (Qiagen Inc.) and following the manufacturer’s instructions with slight modifications. We kept one negative control (water) in each batch of samples to monitor contamination during DNA extraction. To avoid contamination from fecal samples, DNA extraction was carried out in a separate room from PCR replication. All scat samples were first screened for species identification using species-specific primers developed by Bhagavatula & Singh [45] and Thapa et al. [24]. All the samples were processed for genetic screening at CMDN-Laboratory in Kathmandu, Nepal.

### Microsatellite primer selection

We screened variations at 19 loci that were developed for domestic cats [46] and used in previous studies of tigers [24,45]. We optimized use of the primers that were in use on the Indian side of the Terai Arc (Dr. S.P. Goyal: Personal Communication, Wildlife Institute of India) so that our data is comparable to India’s in the future. We selected 10 loci based on expected heterozygosity and variation in allelic diversity. Details on primers used in the study can be found in Thapa et al. [24].

### PCR standardization and genotyping

All PCR standardization and genotyping assay was carried out with field-collected samples (See S1 Appendix, S1 Table). Final consensus genotypes was developed, and details can be found in Thapa et al.[24].

## Data analysis

### Individual tiger identification with camera-trap data

Two investigators independently identified photos to build consensus on individual tiger identity. Individual tigers were identified based on their stripe patterns on their flanks, limbs, tails, and forequarters, and given unique identification numbers, as done in other studies [14,47]. We chose subsets of data from both surveys, representing the core of the CNP, to meet our study objectives for analyzing the datasets from two sources: camera-trap data and fecal DNA markers under SECR framework [4].

### PCR amplification, genotype accuracy and error rates

We used data from the last two PCR runs for standardizing data collection across the runs for all loci across all the tiger-positive samples to calculate PCR amplification rates, genotype accuracy, and error rates (See Table S1 [24],).

### Individual tiger identification with genetic data

We checked for probability of identity for unrelated individuals (P_(ID)_) and siblings (P_(ID)sib_) [24]. We used the minimum criteria of P_(ID)sib_ < 0.010 [29,48] for selecting the minimum number of loci required for individual identification. We used program GenAlEx, version 6.5 [49] to determine the minimum number of individual tigers in the consensus genotype data sets. Any repeatedly matching genotypes were interpreted as recaptures of individual tigers.

### Density estimation

Unlike camera-trapping where detectors are stationary, genetic data can be collected anywhere along the survey routes. While this aspect does not present a problem for traditional capture-recapture analysis methods, the spatially explicit models generally determine detectability based on stationary detectors. Therefore, we needed to assign scat samples collected to a specific discrete detector in space. Therefore, we compared the use of suitable “detectors” at two different scales, the grid versus the segment level. At the grid level, we divided the study area into 1-km^2^ grid cells and used the mid-points of the grid cells to represent the stationary trap location. All DNA collected within that 1 km^2^ grid cell was assigned the location of the center of that cell [50]. At the route-based level, we used every 1-km linear segment as the “trapping device” that accumulated DNA evidence, and the location was represented by the mid-point of each 1-km-long linear segment [30]. Assigning detections collected across a spatial unit (a grid cell or route segment) to a fixed centroid location is a form of partial spatial aggregation, which can influence estimated detection probability and the movement-scale parameter (sigma, σ) if a detector resolution is coarse relative to the species’ spatial scale of movement [51,52]. Our grid-based detector framework followed a general approach developed for unstructured non-invasive sampling data [50]. We selected a 1 km^2^ resolution expected to be small relative to tiger home range movement, our subsequently estimated sigma (σ) values (5,072 m for camera data; 8,443 m for genetic data) confirm that detector resolution was substantially finer than the spatial scale over which individuals were detected, consistent with approach of Lopez-Bao et al. [51] in selecting grid cells small relative to home range size of wolves.

We checked for spatial autocorrelation [53] between grids and segments (1 km^2^ and 1 km) on tiger detections based on Moron’s I statistic in ArcGIS 10.1 (using the default options). We used both SECR-ML and SECR-B approaches with genetic datasets to compare between grid versus segment level “trapping devices”. We considered density estimates to be different when their 95% CI or CrIs (Credible Intervals) did not overlap [54] between the trapping methods.

We used SECR models using the maximum likelihood (SECR-ML) and the Bayesian (SECR-B) approach for estimating densities including models with explicitly modeled heterogeneity in baseline detection probability (g0) and/or the spatial scale parameter (sigma, σ) via two-class finite mixtures, in addition to models incorporating a behavioral response to capture, to account for variation in detectability beyond that inherently inducted by spatial relationship between activity centers and detectors [13,31,55,56]. We used the SECR-ML implemented in program DENSITY version 5.0 [57] and SECR-B implemented in program SPACECAP [58] for both the data sets.

In Program DENSITY version 5.0, we first modeled to select the detection (observational) process as half-normal. Using the selected detection function, we then allowed g_0_ (the capture probability at the hypothetical center of an individual’s home-range) and sigma (sig, a function of the scale of animal movement) to vary using 2-class, finite mixture (h2) to represent heterogeneity, and we included a behavioral response (b). Thus, a half-normal detection function model with 2-class finite mixture of g_0_ and constant sigma would be represented as HN g_0_(h2) sig(.). We used the estimated log likelihood and root-pooled spatial variance (RPSV) of varying integration buffers for determining the appropriate buffer size. We buffered 15 km around the sampling area to represent the probable extent of tiger home-range centers [58]. We carried out the analysis both with and without using a habitat mask. We used a habitat mask due to the presence of distinct blocks of habitat (3,262 km^2^) and non-habitat (3,158 km^2^). We removed the non-habitat (town, city, villages, and agriculture areas: 3,158 km^2^) and assumed uniformity of tiger activity centers over the remaining blocks judged to be suitable. We ranked all the models using sample size adjusted Akaike’s Information Criterion (AICc) and considered all models with ΔAIC_c_ < 2 as competing models. We used model-averaging techniques to determine final density estimates [59]. We report the unconditional variance estimates for the model-averaged estimates. We considered parameters to be different when their 95% CI or CrI did not overlap [32,54] between two data sources.

For the SECR-B approach, for both camera and genetic data sets, we used program SPACECAP version 1.1.0 implemented in R package (version 4.3.3, R Development Core Team, 2021) for estimating tiger density. We used the same grid of hypothetical home-range centers with equally spaced points (n = 7,524), each 1 km apart as done for SECR-ML. This resulted in an area of 3,262 km^2^ of tiger habitat (n = 3,779) over which these activity centers were uniformly distributed. We carried out the analysis both with and without using the habitat mask. For the habitat mask, we removed the 3,158 km^2^ area of settlements (town, city, villages, and agriculture areas) from the hypothetical state space. We used three standard input data files (animal capture locations and dates, trap deployment dates and locations, and the hypothetical activity centers) and assumed a half-normal detection function. We performed 250,000 iterations for the camera data set and 150,000 iterations for the genetic data set, of which the initial 50,000 were discarded as the burn-in period, and we used an augmentation value of 250 individuals (more than five times the expected number of animals). We evaluated the results using the Geweke diagnostic [60] and z-score statistics of |*z*−score| more than 1.6 implying lack of convergence [58]. We considered densities to be different from each other when their 95% CrIs did not overlap [32,54] between two data sources. We produced the pixelated density map showing the estimated tiger densities per pixel of size 1 km^2^ pixel size using ArcGIS 10.1.

We calculated the Coefficient of Variation ($CV=\left(\frac{SE}{mean}\right)*\text{1}\text{0}\text{0}$) as measure of dispersion around mean. This describes the precision of abundance ($\hat{N})$ and density ($\hat{D})$ estimate, expressed as a percentage, to evaluate the relative precision of each estimator for both data sources.

### Population abundance estimation

We used SECR models in maximum likelihood (SECR-ML) and Bayesian (SECR-B) frameworks for estimating tiger abundance including heterogeneity in capture probabilities among individuals in a population [13,31,55]. We used the package “secr” (version 4.6.6) in the R package (version 4.6.6, R Development Core Team, 2021) for both the data sets. We ranked models using sample size adjusted Akaike’s Information Criterion (AIC) [61] and considered all models with ΔAICc < 2 as competing models [59].

We applied the conversion factor for conversed values from SECR models using the Bayesian (SECR-B) implemented in program SPACECAP [58]. We followed Srivathsa et al. [62] used for dholes and Thapa & Kelly [16] used for tigers for calculating the effective sampled area respectively. We used the estimated sigma (σ) value derived from converged SECR-B models and added the buffer as “sigma (σ) × sqrt (5.99)” to the camera trap and genetic data array for estimating effective sampled areas [56]. We also calculated tiger abundance by multiplying the estimated density from SECR models by the respective effective sampled areas [56].

## Results

### Sampling effort and individual tigers captured with camera trap data

We amassed 2,821 trap-nights and obtained 264 identifiable photographs comprising 145 right flanks and 119 left flanks tiger photographs from the core lowland forest of Chitwan National Park. Two researchers matched 100% of all capture events (*n* = 103) and identified 41individuals visually assessed to be 1 year or older (12 males, 16 females, 13 unknown sex) and built capture histories for all identified tigers (Table 1).

**Table 1:** Summary statistics for photographic and genetic capture-recapture data from the core lowland forest in Chitwan National Park, Nepal in 2009 and 2011.

Survey Summary	Cameras	Genetic
Number of Stations (Cameras)/segments (Genetics)*	187	222
Sampling Days	105	42
Sampling Occasions	16	6^#^
Survey Effort	2,821 trap-nights	1,172.5 km of transects
Total number of individuals caught, M_t + 1_	41	31
Total number of captures	103	42
Number of individual animals caught only once	17	17
Number of individual animals caught more than once	24	14
Survey Period	Jan-Mar, 2009	Nov-Dec, 2011

*1 station per 1 km^2^ grid; each segment: 1 km; # repeated survey: 6 days per survey

### Sampling effort and individual tigers identified with genetic data

We walked total of 1173 km on routes and collected 257 scat samples along the designated routes in Chitwan National Park. Using 8 loci genetic assay, we identified 31 individual tigers from scat samples, with PCR amplification success rate of 86.3%, a genotype success rate of 56% and a genotype accuracy rate of 87% [24]. The cumulative P_(ID)_ and P_(ID)sib_ were estimated to be 1.5E-05 and 5.8E-03 (<0.01) respectively We built detection histories for these 31 identified individuals across 42 recapture events based on 8 loci (Table 1).

### Population density and abundance estimation

We computed density estimates (no. of tiger per 100 km^2^) for two types of trapping devices applied to the genetic dataset (1-km^2^ grids versus 1 km route based) and found little difference in density estimates (SECR-ML: 2.18 Vs 2.57; SCR-B: 2.27 Vs 2.12), as the 95% of the CI overlapped substantially (Fig. 2). Moving forward, we used the grid-based trapping device in further analyses for comparability with earlier studies [32] and to our camera trap estimates. A test for spatial autocorrelation between the trapping devices (grids), tiger detection was highly clustered across the lowland areas (Z score > 3.84, *p<*=0.00).

**Fig 2:**
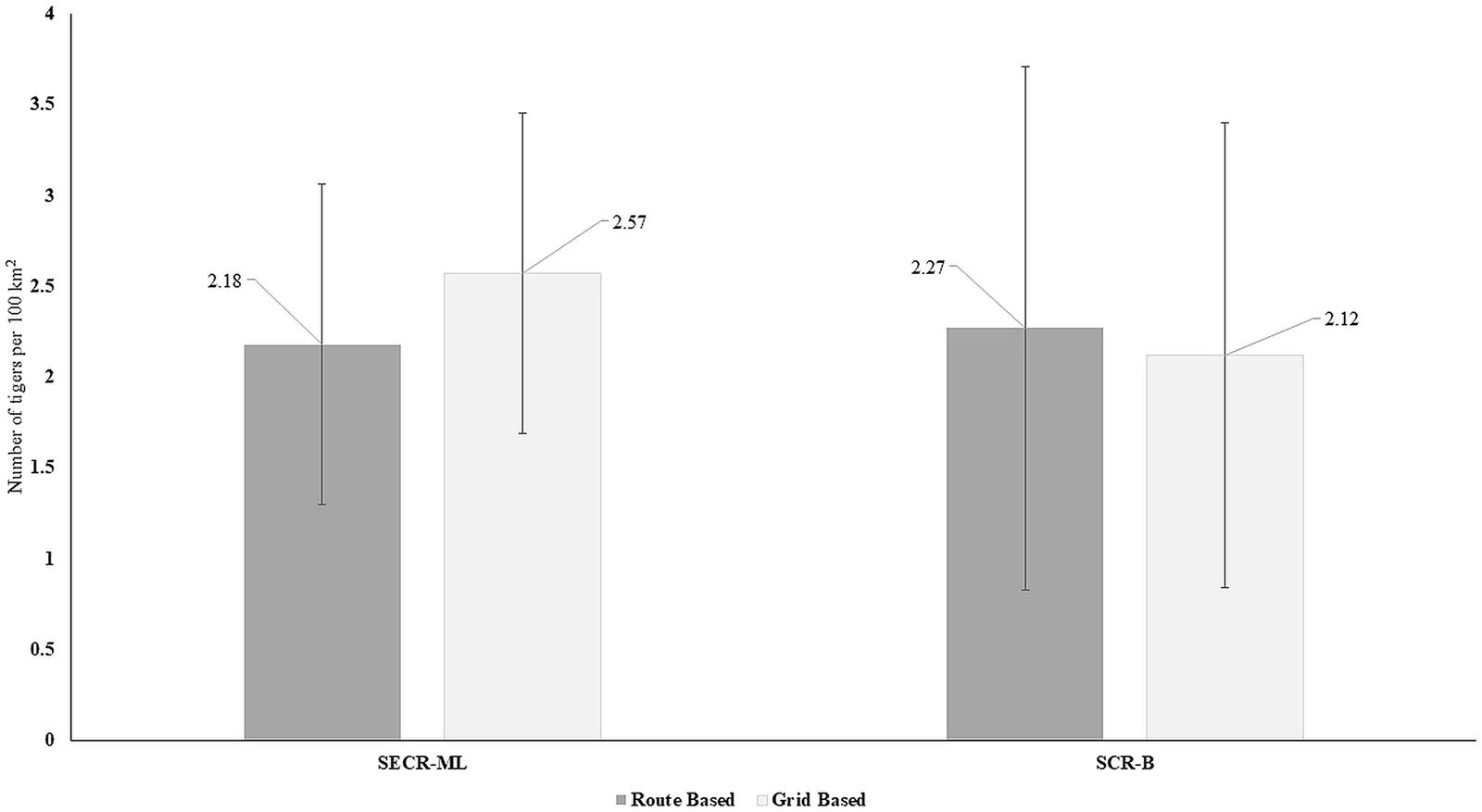
Density estimates using genetic sampling and 2 types of “stationary trapping devices” (grid versus 1 km segment levels) with spatially explicit maximum likelihood (SECR-ML) and Bayesian (SECR-B) models. Error bars represent 95% CIs or CrIs.

We selected the appropriate buffer sizes of 15,000 m and 24,000 m based on estimated log likelihood and RPSV for camera and fecal DNA data sets, respectively in Program DENSITY (Table 2). For the camera-trap data, the top model supported the half normal detection function with constant detectability at home-range center (g_o_) and a constant response in spatial scale over which detection declined (function of movement, σ) (Table 2). There were 3 competing models that included effects of heterogeneity and behavior on g_o_ and σ. The model-averaged density estimates for the camera-trap dataset based on SECR-ML was 2.55 (SE 0.59) tigers per 100 km^2^ with habitat mask (Fig 3) and 1.89 (SE 1.47) tigers per 100 km^2^ without the habitat mask.

**Table 2:** Model selection results from tiger density estimates using photographic and genetic capture-recapture data from the lowland forest of Chitwan National Park, implemented in Program DENSITY using the half normal detection function. Akaike’s Information Criterion adjusted for small sample size is represented by AICc, w_i_ represents Akaike weight, $\hat{D}$ is the estimated density (tigers per 100 km^2^), and SE is the standard error. The model average density estimates, and standard errors are given in bold.

Data Type	Model	AIC_c_	∆AIC_c_	*w*_i_	$\hat{𝐃\;}$(SE)
Cameras	g_0_[.]s[.]	1895.61	0	0.28	2.56 ± 0.47
	g_0_[bh2]s[h2]	1896.35	0.74	0.19	2.65 ± 0.56
	g_0_[b]s[.]	1897.08	1.47	0.13	2.72 ± 0.55
	g_0_[.]s[bh2]	1897.55	1.94	0.11	2.99 ± 0.75
	g_0_[.]s[b]	1898.01	2.4	0.08	2.61 ± 0.59
	g_0_[bh2]s[bh2]	1898.77	3.16	0.06	2.93 ± 0.84
	g_0_[h2]s[b]	1899.01	3.4	0.05	2.52 ± 0.54
	g_0_[bh2]s[.]	1899.17	3.56	0.05	2.77 ± 0.58
	**Model averaged results from top 8 models**				**2.55 ± 0.59**
Genetic	g_0_[.]s[b]	671.01	0	0.42	2.68 ± 0.81
	g_0_[b]s[b]	672.26	1.25	0.23	2.59 ± 0.78
	g_0_[b]s[.]	673.44	2.43	0.13	2.91 ± 0.90
	g_0_[.]s[.]	673.6	2.59	0.12	3.52 ± 1.0
	g_0_[h2]s[b]	676.74	5.73	0.02	2.67 ± 0.81
	**Model averaged results from top 5 models**				**2.57 ± 0.88**

g_o_ is the capture probability at home-range center, σ s is the spatial scale over which detection declines from the home range center, h_2_ is the 2-class finite mixture probability for heterogeneity, b: behavioral response.

**Fig 3:**
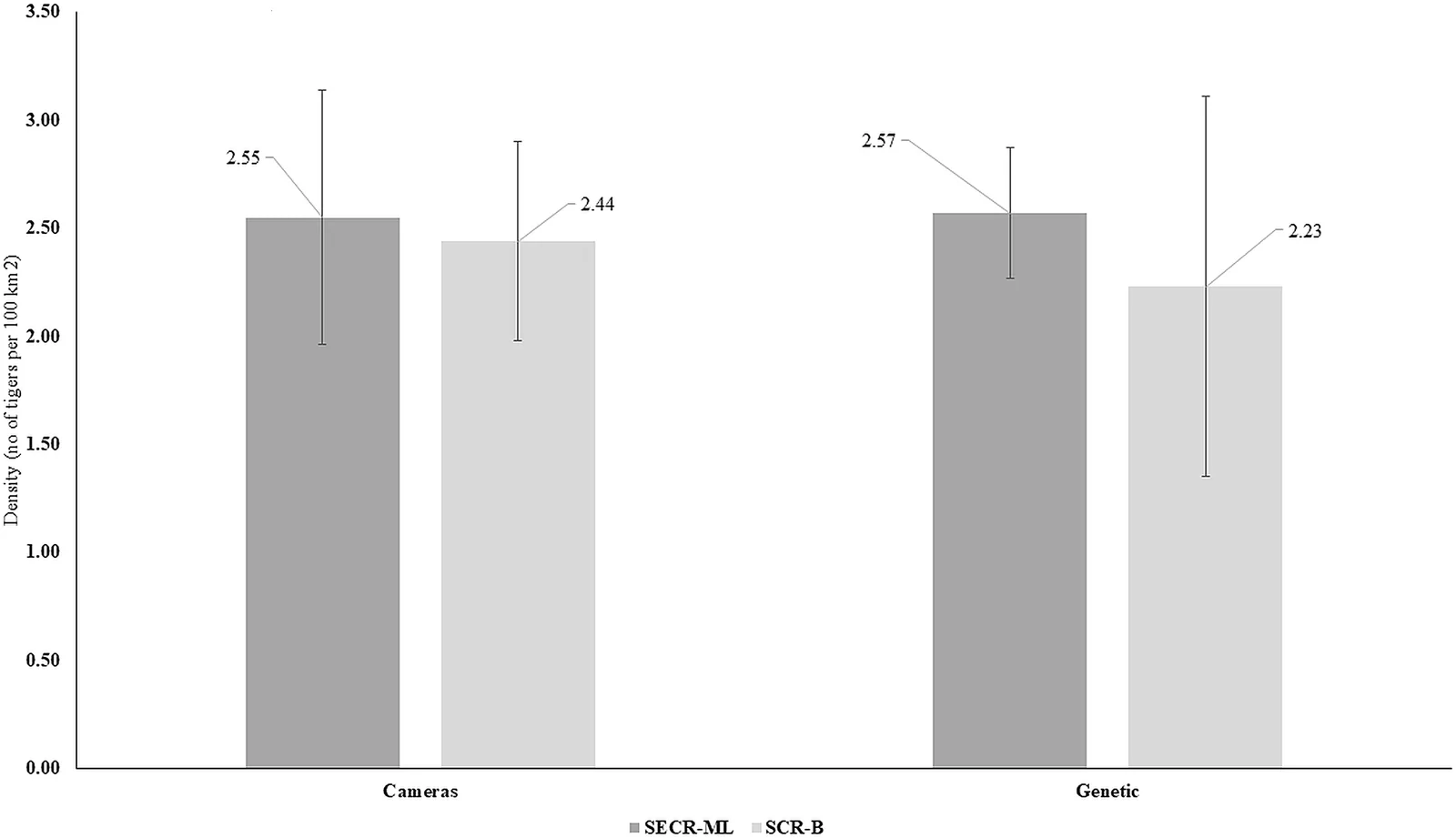
Density estimates of tigers per 100 km^2^ with photographic and genetic data implemented with spatially explicit maximum likelihood (SECR-ML) and Bayesian (SECR-B) models. Error bars represent 95% CIs or CrIs.

Model selection in SECR-ML with fecal DNA data supported the hazard rate detection function with the top model including constant detectability at home-range center, g_o_, and a behavioral response in spatial scale (σ) (Table 2). There was one competing model with some support for behavioral response in detectability. The model-averaged density estimates for the genetic dataset based on SECR-ML were 2.57 (SE 0.88) tigers per 100 km^2^ with habitat mask and 2.18 (SE 0.88) tigers per 100 km^2^ without habitat mask.

Using the SECR-B analysis implemented in SPACECAP, all model parameters converged based on Geweke diagnostic statistics with z scores less than 1.6 [58] for both data sets. For the camera-trap data, we obtained the posterior density estimates of 2.44 (SE 0.30) tigers per 100 km^2^ with habitat mask (Fig 3) and 1.77 (SE 0.23) tigers per 100 km^2^ (Table 4) without habitat mask. For the genetic data, we obtained similar posterior density estimates of 2.23 (SE 0.46) tigers per 100 km^2^ (Table 3) with habitat mask (Fig.3) and 1.41 (SE 0.29) tigers per 100 km^2^ without habitat mask. The resulting pixelated density maps show the relative distribution of animal densities across the home range centers (Fig.4), with variation observed across floodplain, riverine and sal forest habitat in Chitwan National Park.

**Table 3:** The posterior summaries from the Bayesian spatially explicit capture recapture (SECR-B) models, and the tiger density estimates, implemented in Program SPACECAP for tigers from the lowland forest of Chitwan National Park for photographic and fecal datasets. A Geweke statistic |z score| of greater than 1.6 implies lack of convergence.

Data Type	Parameter	Posterior Mean	Posterior SD	95% Lower HPD Level	95% Upper HPD Level	Geweke’s Statistics |z score|
Cameras	σ	5072.14	345.02	4402.63	5752.74	0.1895
	*λ*_0_	0.03	0.00	0.02	0.04	0.3647
	Ψ	0.27	0.04	0.19	0.36	−0.4854
	*N*	79.39	9.95	60.00	98.00	−0.5945
	*D*	0.02	0.00	0.02	0.03	
Genetic	σ	8442.86	1298.95	6218.87	11038.75	0.1223
	*λ*_0_	0.01	0.00	0.00	0.01	0.7222
	*B*	2.96	0.35	2.30	3.65	−0.9012
	Ψ	0.26	0.06	0.15	0.38	−0.9543
	*N*	72.40	14.91	45.00	101.00	−0.9919
	*D*	2.23	0.46	1.44	3.17	
	*p1*	0.01	0.00	0.00	0.01	
	*p2*	0.11	0.03	0.06	0.17	

Sigma (σ) represents the range parameter of the species while lambda (λ_0_) is the intercept of the expected encounter frequency. Psi (Ψ) is the ratio of the number of animals present within the state space, S, to the maximum allowable number. Nsuper (N) is the number of activity centers in S. Density (D, tigers per 100 km^2^) is Ns divided by S.

**Table 4:** Summary of model selection results from tiger population abundance estimates using photographic and genetic capture recapture data for the tiger populations using spatially explicit capture/recapture in SECR packages in R. AICc represents Akaike’s Information Criterion, corrected for the small sample size. The ΔAICc is the difference in AIC values between the top model and i^th^ model in the set. Weight of support for each model is shown by the AICcwt weights (ω_i_).

Data Type	Model	AIC	AICc	ΔAICc	*w*_i_
Cameras	g0 ~ h2 σ ~ 1 pmix~h2	1037.114	1038.225	0	1
	g0 ~ h2 σ ~ B pmix~h2	1831.282	1832.997	794.772	0
	g0 ~ h2 σ ~ b pmix~h2	1855.814	1857.528	819.303	0
	g0 ~ h2 σ ~ bk pmix~h2	1856.61	1858.324	820.099	0
	g0 ~ h2 σ ~ Bk pmix~h2	1862.088	1863.802	825.577	0
	g0 ~ h2 σ ~ T pmix~h2	1865.286	1867.001	828.776	0
Genetic	g0 ~ 1 σ ~ h2 pmix~h2	556.148	557.687	0	1
	g0 ~ 1 σ ~ 1	578.234	578.663	20.976	0
	g0 ~ 1 σ ~ bk	817.81	818.699	261.012	0
	g0 ~ 1 σ ~ T	839.007	839.896	282.209	0
	g0 ~ 1 σ ~ Bk	842.295	843.184	285.497	0
	g0 ~ 1 σ ~ b	842.367	843.256	285.569	0
	g0 ~ 1 sigma~B	842.543	843.432	285.745	0

g0: detection probability; σ: space range; T: time trend; B:transient response; h2: two-class mixtures, pmix: probability of belonging to mixture 1; bk: behavioral response to capture; b: a null and permanent global learned response model, bk: a permanent detector-specific learned response model.

**Fig 4:**
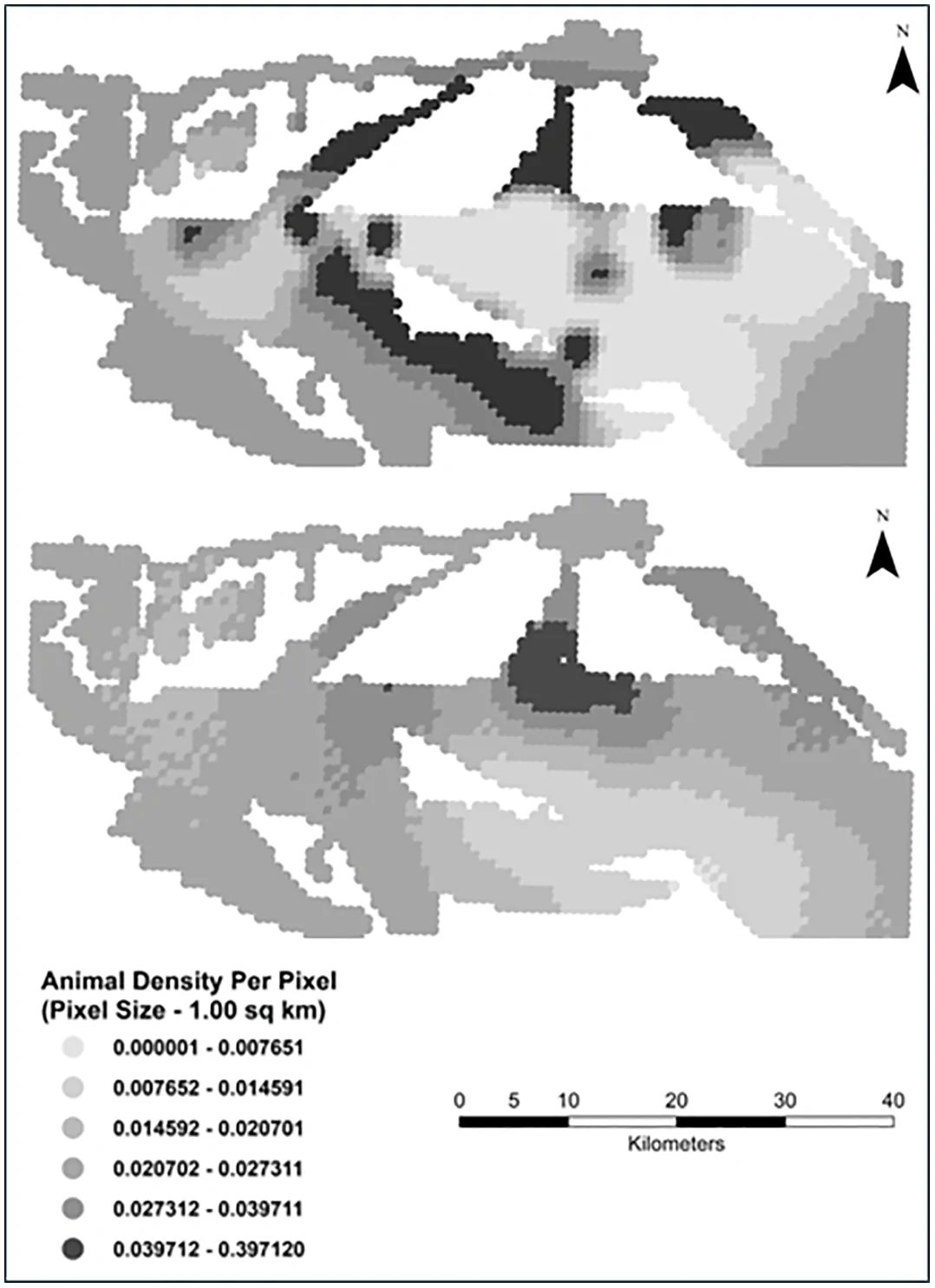
A pixelated density map showing the relative tiger densities per pixel of size 1 km^2^ using (above) camera-trap data and (below) genetic data, prepared in ArcGIS 10.1.

SECR-ML estimates based the top model that included heterogeneity and behavior effects (g0 ~ h2 σ ~ 1 pmix~h2**,** AICwt = 1, Table 4) for camera trap data and used a habitat mask, estimated an abundance of 65.6 (SE 7.9) tigers. The top model for the genetic dataset (g0 ~ 1 σ ~ h2 pmix~h2) estimated g0 = 0.08 (SE 0.01) and σ = 2,692 (SE 211) m, indicating detection probability at the activity center and spatial scale of movement. For genetic detectors based on behavior in the top model (g0 ~ 1 σ ~ h2 pmix~h2, AICwt = 1, Table 4) using the segment-based trapping approach estimated abundance of 88.3 (19.26) tigers. The top model for the genetic dataset estimated g0 = 0.05 (SE 0.01) and σ = 2,755 (SE 373) m, indicating detection probability at the activity center and spatial scale of movement.

Using the SECR-B posterior density estimates multiplied by the effective sample area, we estimated tiger abundances ($\hat{N}$(SE)) to be approximately 64 (SE 7.90) for camera trap datasets and 93 (SE 19.08) for genetic data sets (Fig 5) respectively. Among the estimators, population estimates were comparable between SECR-ML and SECR-B for respective data sources as 95% CI and CrI did not overlap.

**Fig 5:**
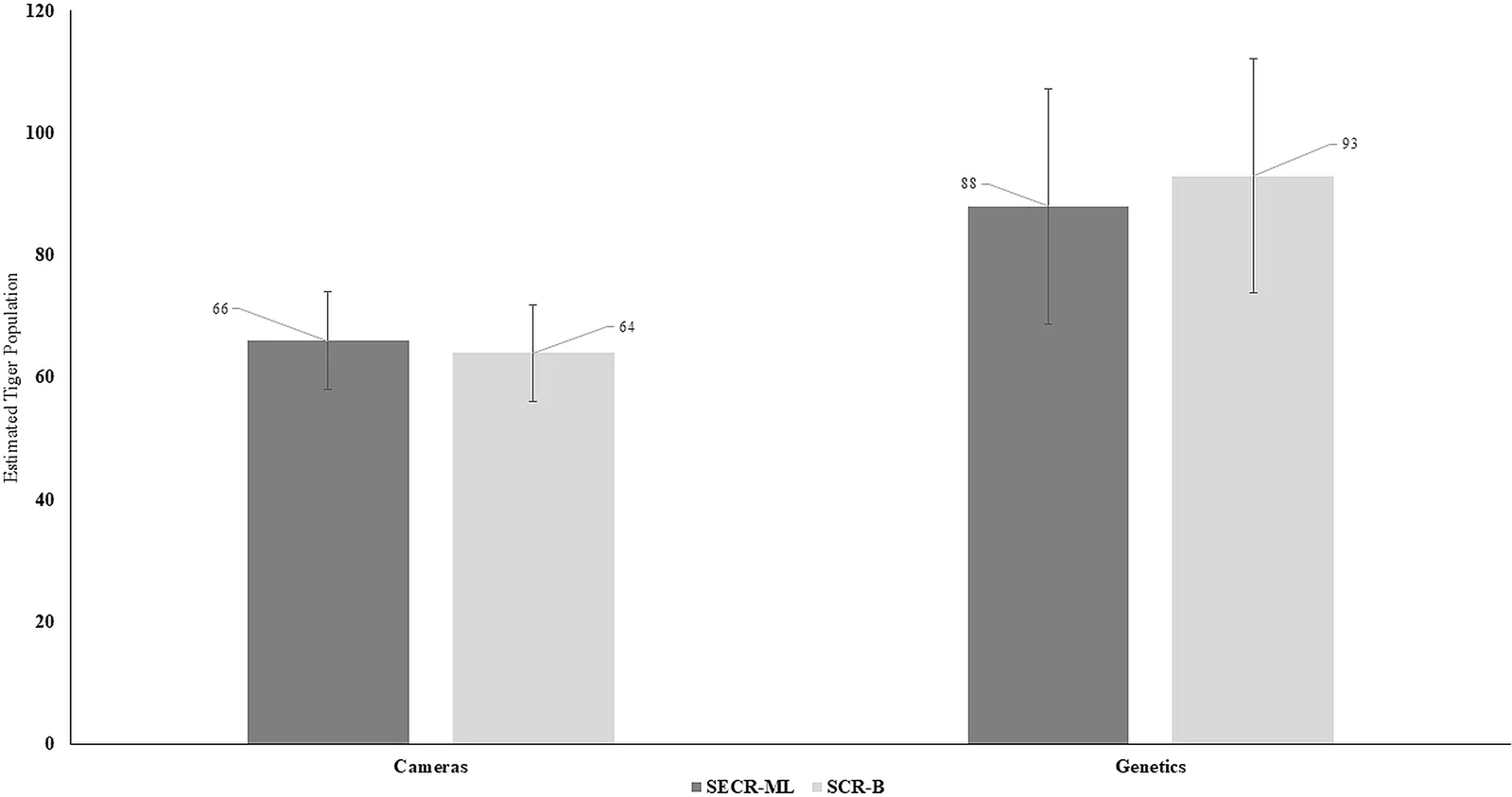
Abundance estimates via spatially explicit mark-recapture of tigers in Chitwan National Park, Nepal using a photographic versus a genetic capture-recapture framework implemented in a maximum likelihood (SECR-ML) and Bayesian (SECR-B) framework.

## Discussion

This is the first study using non-invasive genetic sampling to empirically estimate the abundance and density of tigers across the Terai Arc Landscape, generating estimates that we present alongside those from a well-established camera trap protocol [13,30–32] conducted in the same area. The main findings of this study were: 1) the core lowland forest area of CNP that overlaps with both survey held minimum of 31–41 individuals, and the density estimates ranged from 2.27 to 2.44 tigers per 100 km^2^; 2) abundance estimates between analytic approaches (SECR-ML and SECR-B) were similar within each data source; and 3) estimates were broadly comparable between the two data sets, though because sampling method and survey year are fully confounded in this design, this comparability cannot be used to infer either population stability or the relative performance of the two methods.

Careful consideration is needed when conducting fecal sampling to reduce DNA degradation, which can result from long delays between field collection and sample processing [63]. With our field-sampling design, we collected scat samples less than 7 days old and repeated sampling every 7 days, enabling us to achieve a high sample size (n = 257) within a relatively short duration while still fulfilling the closed population assumption. Improved field protocols, stringent laboratory conditions, an optimized panel of 8 polymorphic markers, and sufficient PCR replicates (3 to 6) enabled us to reliably identify individuals from field-collected samples with varying DNA quality and quantity. Our methods increased our PCR amplification rate, reduced genotyping errors, and yielded more reliable genotypes than in other recent non-invasive genetic sampling studies of felids based on a similar number of loci (61.2% for jaguars *P. onca* [44]; 70% for Bengal tigers *P. tigris tigris* [64]; and 53% for snow leopards *Panthera uncia* [65],). The criterion of P(ID)sib < 0.010 [29,48] was sufficiently high in our optimized panel of loci for resolving relationships between related individuals with adequate statistical power.

In addition to the coverage gaps discussed above, our use of centroid-based detectors for genetic samples introduces a distinct and separate potential source of bias: partial spatial aggregation of detections to a fixed grid or segment centroid [51,52]. We selected a resolution expected to be fine relative to tiger movement scale, and our σ estimates support this expectation, but we did not formally quantify the magnitude of any residual aggregation bias, as done for wolves by Milleret et al. [52]. We recommend that future non-invasive genetic SCR studies of tigers explicitly evaluate detector resolution against σ using simulation, rather than assuming aggregation effects are negligible by design alone. Unlike Russell et al.[50], we did not incorporate grid-cell-specific search effort as a covariate on detection probability. Search routes were pre-defined transects of similar character (firelines, roads, and trails) surveyed by the same three-observer teams across all sampling blocks, and transect length was reasonably consistent among grid cells; however, we acknowledge that residual variation in effort among cells was not explicitly modeled, and future applications of this method should incorporate cell-specific effort as a covariate where transect coverage varies more substantially.

However, a notable limitation of the genetic dataset is the low rate of individual recapture: only 14 of 31 (45%) genetically identified tigers were recaptured at all, and the number recaptured at a different detector (i.e., spatial recaptures) was still smaller. Sigma, σ, the parameter describing the spatial scale of animal movement, is estimated directly from the distances between an individual's recapture locations. Sparse spatial recaptures reduce the precision of σ, and can also bias it — for example, if most recaptures occur at the same or an adjacent segment, σ may be underestimated, which would in turn understate the effective sampled area and could inflate density estimates. This is consistent with the wider coefficient of variation we observed for genetic density estimates (SECR-ML CV ≈ 34%) relative to camera-trap estimates (SECR-ML CV ≈ 23%) and is a more direct constraint to precision than raw sample size alone. Given this, we recommend that future non-invasive genetic surveys of tigers and other rare carnivores be explicitly designed to increase the probability of spatial recapture — for example, through denser or more frequently repeated transects, longer survey duration, or a full-coverage grid design rather than linear routes confined to trails — rather than prioritizing total sample size in isolation.

A related limitation concerns the spatial configuration of our sampling effort. Camera and transect placement targeted known tiger travel routes and sign concentrations to maximize per-unit detection probability, following established survey protocol for this species [14,42], rather than a systematic or grid-based array. This approach is standard practice for maximizing tiger detection rates in a landscape where trails and routes concentrate movement, but it produced substantial unsampled gaps in the interior of the study area (Fig 1). If any tiger home ranges were centered entirely within these gaps, those individuals would have had near-zero probability of detection, which can lead to underestimation of both abundance and density and violates the SECR assumption that all activity centers within the state space have some non-zero detection probability. The resulting pixel-level density surfaces (Fig 4) are therefore best interpreted as reflecting detection intensity concentrated near trails and transect routes, rather than a complete representation of tiger spatial use across the full landscape.

This spatial-sampling limitation also helps explain a further discrepancy in our results: although the overall average density estimates converged closely between the camera-trap and genetic data sets, the pixel-level density surfaces (Fig 4) diverged substantially in their spatial pattern. We attribute this primarily to differences in the estimated detection parameters between the two data types, σ differed considerably between cameras (5,072 m) and genetic data (8,443 m), and λ_0_ differed by roughly an order of magnitude, combined with the differing spatial geometry of point detectors (cameras) versus linear detectors (transects). These factors directly shape where density is predicted to concentrate on a pixelated surface, independent of whether the underlying tiger distribution truly differed between the two data sets. We therefore caution against interpreting the fine-scale spatial pattern in Fig 4 as an accurate representation of true tiger distribution; the overall average density and abundance estimates, rather than the pixel-level maps, are the more robust and directly comparable outputs of this study.

The behavioral response detected in the genetic detection model (g0 ~ 1 σ ~ h2 pmix~h2 with a behavioral term supported) may reflect scent-marking behavior, whereby tigers preferentially reuse scent-marked travel routes and latrine sites for scat deposition, increasing the likelihood of repeated detection at previously used segments. This contrasts with camera-trap detection, which is passive and not directly linked to the animal's own marking behavior, and may likely explain why a comparable behavioral effect was not similarly supported in the top camera-trap model.

Our sampling efforts showed a higher relative probability of capturing tigers present in the sampled area for cameras (81%) than for genetics (66%), but survey length was longer for cameras than genetics, 105 versus 42 days, respectively. This difference likely reflects the longer survey duration and sampling occasions for the camera trap survey rather than an inherent difference in tiger detectability between two sampling methods. Point estimates from both data sources do not represent the total estimated population size for CNP during that time [66] but represent estimates from the lowland forest in the core areas of CNP. Our results from both analytical platforms (SECR-ML and SECR-B) produced similar results, with lower abundance estimates for cameras than for genetic data, but CIs overlapped. However, estimates were similar for density estimates (SECR-ML vs SECR-B) used irrespective of data sources. All the models produced reasonably precise estimates of population abundance, with coefficients of variation (CV) < 16%. The similar CV (< 12%) were produced by both approach (SECR-ML and SECR-B) which have been shown to produce reasonable estimates of population size with camera trap data in the past [37,67]. Abundance estimates from camera-trap versus genetic data were similar, suggesting broadly comparable density estimates across the two sampling periods (2009 versus 2011) in the sampled core lowland forest of CNP.

We did not perform a sensitivity analysis on the robustness of population parameter estimates [37] due to changes in recapture rates; however, the use of the maximum number of loci (8 loci in this study) produced stable estimates under likelihood and Bayesian estimator against the threshold of 6 loci based on P_ID(sibs)_ [48]. We note that the contribution of tiger cubs to the genetic sample size is likely negligible because we only included large scat sizes [68] and did not include small scats that may have been from tiger cubs within the collected sample size. Thus, the likelihood of tiger cub captured and counted in population estimates is low, and our estimates likely reflect population size for adult and sub-adult tigers only [14].

Of the density estimators used in this study, tiger densities from SECR-ML and SECR-B models were similar [58]. Point estimates were similar, and the 95% CI and CrIs overlapped for both data sources. However, SECR-B models were more precise (ave CV = 7%) than SECR-ML (ave CV = 28%), possibly due to a few competing SECR-ML models and the inherent model uncertainties in the model-averaged estimates [55,59]. Given the caveats above regarding the spatial recapture rates, detector configuration, and the differing survey timeframes and methods, we emphasize that overall density and abundance point estimates between the two independent data sources should not be interpreted as population stability between two-time frames, nor evidence as evidence that two methods converge in general.

Advanced analytical frameworks employing spatially explicit capture-recapture [56] can give us more insight into the usefulness of both types of non-invasive data sources. Contingent upon a field design that permits SECR analysis and a high degree of certainty in individual identification with fecal DNA samples, non-invasive genetic sampling likely can be improved by increasing sample size to obtain more recaptures-more spatial recaptures-and this approach should be adopted instead of the adhoc survey techniques used in many genetic studies [45,69]. Repeated transect surveys for scat give us more encounter occasions and potential for spatial re-captures, which are necessary for improved SECR modeling.

This study highlights the potential use of a non-invasive genetic approach in population monitoring across the core area of TAL and for comparing population estimates from camera-trapping to improve and complement the past efforts [6,66,70]. Combining camera-trapping and genetic data, ideally collected concurrently in the same time period to permit a valid comparison, can be beneficial in improving inference about population demographic parameters as done for tigers [30], for Jaguar [32], and for snow leopard [39]. However, the choice of appropriate non-invasive techniques is contingent upon cost (laboratory), logistics, and expertise [71]. In the TAL, successful population monitoring programs like the Tiger Census [72] and the Nepal Tiger Genome Project [24] can be integrated in analyses within an emerging SECR model framework [10,12,13,30–32]. The overall findings suggest that both camera trapping and genetic sampling can produce comparable density, and abundance estimates when properly implemented, and that integrating data from two both sources-collected concurrently and with attention to spatial recaptures rates and detector coverage and integrating both data sets in one single analytical platform could be way forward. The new analytical techniques could be beneficial for improving population and density estimates regarding the status of tiger populations in TAL and beyond.

## Supporting information

S1 Appendix PCR Standardization and Genotyping. (DOCX)

S1 Table Summary of PCR amplification success, genotyping accuracy and genotyping error rates for 8 microsatellite loci for all processed tiger positive samples (n = 257) in Chitwan National Park: PCR, % polymerase chain reaction amplification success; GA, % genotyping accuracy; ADO, % allelic dropout; FA, % false alleles. (DOCX)
