# Supplementary material for: Tiger abundance and density estimates from non-invasive sources: Camera-trap and genetic capture-recapture analyses
Source: PLoS One. 2026 Nov 4;21(11):e0361369. doi: 10.1371/journal.pone.0361369 (PMC13734577; doi:10.1371/journal.pone.0361369)
Supplement: Supplementary file 1 [file pone.0361369.s001.docx]

**S1 Appendix: PCR Standardization and Genotyping**

All PCR standardization was carried out with field-collected samples (Thapa et al., 2018). For all standardizations, amplification was carried out in 7-µl reaction volumes containing 3.5 µl Qiagen Mastermix and 0.07 µl of Q solution, forward and reverse primers for all the microsatellite loci mixed to final standard concentration: 0.4 µM FCA205, 0.2µM FCA391, 0.07µM PttD5, 0.14µM FCA232, 0.07µM FCA304, 0.30µM F85, 0.14µM FCA441, 0.07µM PttA2, 0.09µM FCA043 and 0.49µM F53 and 2.50µl of DNA extract. The PCR conditions for microsatellites were as follow: The temperature regime included an initial denaturation step (95^◦^C for 15 min); initial touchdown PCR was performed for 10 cycles with denaturation (94 ^◦^C for 30s), annealing (62^◦^C for 90s) reduced by 0.5◦C in each succeeding cycle and extension (72^◦^C for 60s); followed by 25 cycles of denaturation (94^◦^C for 30s), annealing (57^◦^C for 90s) and extension (72^◦^C for 60s) followed by a final extension (72^◦^C for 10 min), and cooling to 4^◦^C in an MJ Research PTC-225 thermal cycler. 0.7µl of amplified product was added to 0.3µl of LIZ-500 size standard and then the genotyping assay was run in an automated ABI 310 genetic analyzer (Applied Biosystems). Microsatellite alleles were scored using GENEMAPPER-4.1 (Applied Biosystems). Each sample was amplified 4-6 times in a multitube approach. To finalize the consensus genotypes, a multitube approach was used where at least three identical homozygote PCR results were required for scoring homozygote genotypes, and each allele had to be observed in two independent PCRs to score a heterozygous genotype (Wultsch et al. 2014).

**References:**

Thapa, K., Manandhar, S., Bista, M., Shakya, J., Sah, G., Dhakal, M., Sharma, N., Llewellyn, B., Wultsch, C. & Waits, L. P. (2018) Assessment of genetic diversity, population structure, and gene flow of tigers (Panthera tigris tigris) across Nepal's Terai Arc Landscape. PloS one 13: e0193495

Wultsch, C., L. P. Waits, and M. J. Kelly. 2014. Noninvasive individual and species identification of jaguars (Panthera onca), pumas (Puma concolor) and ocelots (Leopardus pardalis) in Belize, Central America using cross-species microsatellites and faecal DNA. Molecular Ecology Resources:n/a-n/a.
