# Supplementary material for: Tiger abundance and density estimates from non-invasive sources: Camera-trap and genetic capture-recapture analyses
Source: PLoS One. 2026 Nov 4;21(11):e0361369. doi: 10.1371/journal.pone.0361369 (PMC13734577; doi:10.1371/journal.pone.0361369)
Supplement: Supplementary file 2 [file pone.0361369.s002.docx]

**Supplementary materials**

**S1 Table**: Summary of PCR amplification success, genotyping accuracy and genotyping error rates for 8 microsatellite loci for all processed tiger positive samples (n = 257) in Chitwan National Park: PCR, % polymerase chain reaction amplification success; GA, % genotyping accuracy; ADO, % allelic dropout; FA, % false alleles.

| **S.No** | **Locus** | **Chitwan National Park Population (n=257)** | | | |
| --- | --- | --- | --- | --- | --- |
|  |  | PCR | GA | ADO | FA |
| 1 | FCA391 | 87.58 | 89.47 | 0.47 | 12.60 |
| 2 | PttD5 | 91.69 | 91.99 | 1.04 | 8.01 |
| 3 | FCA232 | 82.79 | 80.17 | 0.62 | 25.51 |
| 4 | FCA304 | 93.95 | 90.25 | 0.00 | 8.66 |
| 5 | FCA043 | 95.22 | 88.97 | 0.00 | 11.03 |
| 6 | F53 | 69.75 | 81.90 | 0.80 | 18.10 |
| 7 | F85 | 75.48 | 85.07 | 0.00 | 15.32 |
| 8 | FCA441 | 94.27 | 85.76 | 0.63 | 14.24 |
|  | Mean | 86.34 | 86.70 | 0.45 | 14.18 |
|  | SD | 9.53 | 4.19 | 0.40 | 5.68 |
